# A picture is worth a thousand words: maps of HIV indicators to inform research, programs, and policy from NA-ACCORD and CCASAnet clinical cohorts

**DOI:** 10.7448/IAS.19.1.20707

**Published:** 2016-04-04

**Authors:** Keri N Althoff, Peter F Rebeiro, David B Hanna, Denis Padgett, Michael A Horberg, Beatriz Grinsztejn, Alison G Abraham, Robert Hogg, M John Gill, Marcelo J Wolff, Angel Mayor, Anita Rachlis, Carolyn Williams, Timothy R Sterling, Mari M Kitahata, Kate Buchacz, Jennifer E Thorne, Carina Cesar, Fernando M Cordero, Sean B Rourke, Juan Sierra-Madero, Jean W Pape, Pedro Cahn, Catherine McGowan, for the North American Aids Cohort Collaboration on Research and Design (na-Accord) and the Caribbean, Central and the South America Network for Hiv Epidemiology (ccasanet)

**Affiliations:** 1 Department of Epidemiology, Johns Hopkins Bloomberg School of Public Health, Baltimore, MD, USA; 2 Department of Medicine, Vanderbilt University, Nashville, TN, USA; 3 Department of Epidemiology and Population Health, Albert Einstein College of Medicine, Bronx, NY, USA; 4 Department of Internal Medicine, Infectious Disease Service, Instituto Hondureño de Seguridad Social, Tegucigalpa, Honduras; 5 Mid-Atlantic Permanente Research Institute, Kaiser Permanente Mid-Atlantic States, Rockville, MD, USA; 6 Evandro Chagas National Institute of Infectious Diseases, Fiocruz, Rio de Janeiro, Brasil; 7 British Columbia Centre for Excellence in HIV/AIDS, Vancouver, British Columbia, Canada; 8 Department of Medicine, University of Calgary, Calgary, Alberta, Canada; 9 Departamento de Medicina, Facultad de Medicina, Universidad de Chile and Fundación Arriarán, Santiago, Chile; 10 Department of Internal Medicine, Universidad Central del Caribe, Bayamón, Puerto Rico, USA; 11 Department of Medicine, Sunnybrook Health Sciences Centre, University of Toronto, Toronto, ON, Canada; 12 Epidemiology, Basic Science Program, Division of AIDS, National Institute of Allergy and Infectious Diseases, Rockville, MD, USA; 13 Department of Medicine, University of Washington, Seattle, WA, USA; 14 Division of HIV/AIDS Prevention, Centers for Disease Control and Prevention, Atlanta, GA, USA; 15 Department of Ophthalmology, Johns Hopkins University School of Medicine, Baltimore, MD, USA; 16 Investigaciones Clínicas, Fundación Huésped, Buenos Aires, Argentina, USA; 17 HIV Research Group, Instituto de Medicina Tropical Alexander von Humboldt, Universidad Peruana Cayetano Heredia, Lima, Perú; 18 Department of Psychiatry, University of Toronto, Toronto, Ontario, Canada; 19 Departamento de Infectología, Instituto Nacional de Ciencias Medicas y Nutrición Salvador Zubirán, Mexico DF, Mexico; 20 Le Groupe Haïtien d'Etude du Sarcome de Kaposi et des Infections Opportunistes, Port-au-Prince, Haiti and Department of Medicine, Weill Cornell Medical College, New York, NY, USA

**Keywords:** Map, HIV indicators, CD4 T-lymphocyte count, retention in care, antiretroviral therapy, HIV RNA suppression, North America, Central America, South America, implementation science

## Abstract

**Introduction:**

Maps are powerful tools for visualization of differences in health indicators by geographical region, but multi-country maps of HIV indicators do not exist, perhaps due to lack of consistent data across countries. Our objective was to create maps of four HIV indicators in North, Central, and South American countries.

**Methods:**

Using data from the North American AIDS Cohort Collaboration on Research and Design (NA-ACCORD) and the Caribbean, Central, and South America network for HIV epidemiology (CCASAnet), we mapped median CD4 at presentation for HIV clinical care, proportion retained in HIV primary care, proportion prescribed antiretroviral therapy (ART), and the proportion with suppressed plasma HIV viral load (VL) from 2010 to 2012 for North, Central, and South America. The 15 Canadian and US clinical cohorts and 7 clinical cohorts in Argentina, Brazil, Chile, Haiti, Honduras, Mexico, and Peru represented approximately 2–7% of persons known to be living with HIV in these countries.

**Results:**

Study populations were selected for each indicator: median CD4 at presentation for care was estimated among 14,811 adults; retention was estimated among 87,979 adults; ART use was estimated among 84,757 adults; and suppressed VL was estimated among 51,118 adults. Only three US states and the District of Columbia had a median CD4 at presentation >350 cells/mm^3^. Haiti, Mexico, and several states had >85% retention in care; lower (50–74%) retention in care was observed in the US West, South, and Mid-Atlantic, and in Argentina, Brazil, and Peru. ART use was highest (90%) in Mexico. The percentages of patients with suppressed VL in the US South and Northeast were lower than in most of Central and South America.

**Conclusions:**

These maps provide visualization of gaps in the quality of HIV care and allow for comparison between and within countries as well as monitoring policy and programme goals within geographical boundaries.

## Introduction

**Figure 1:**
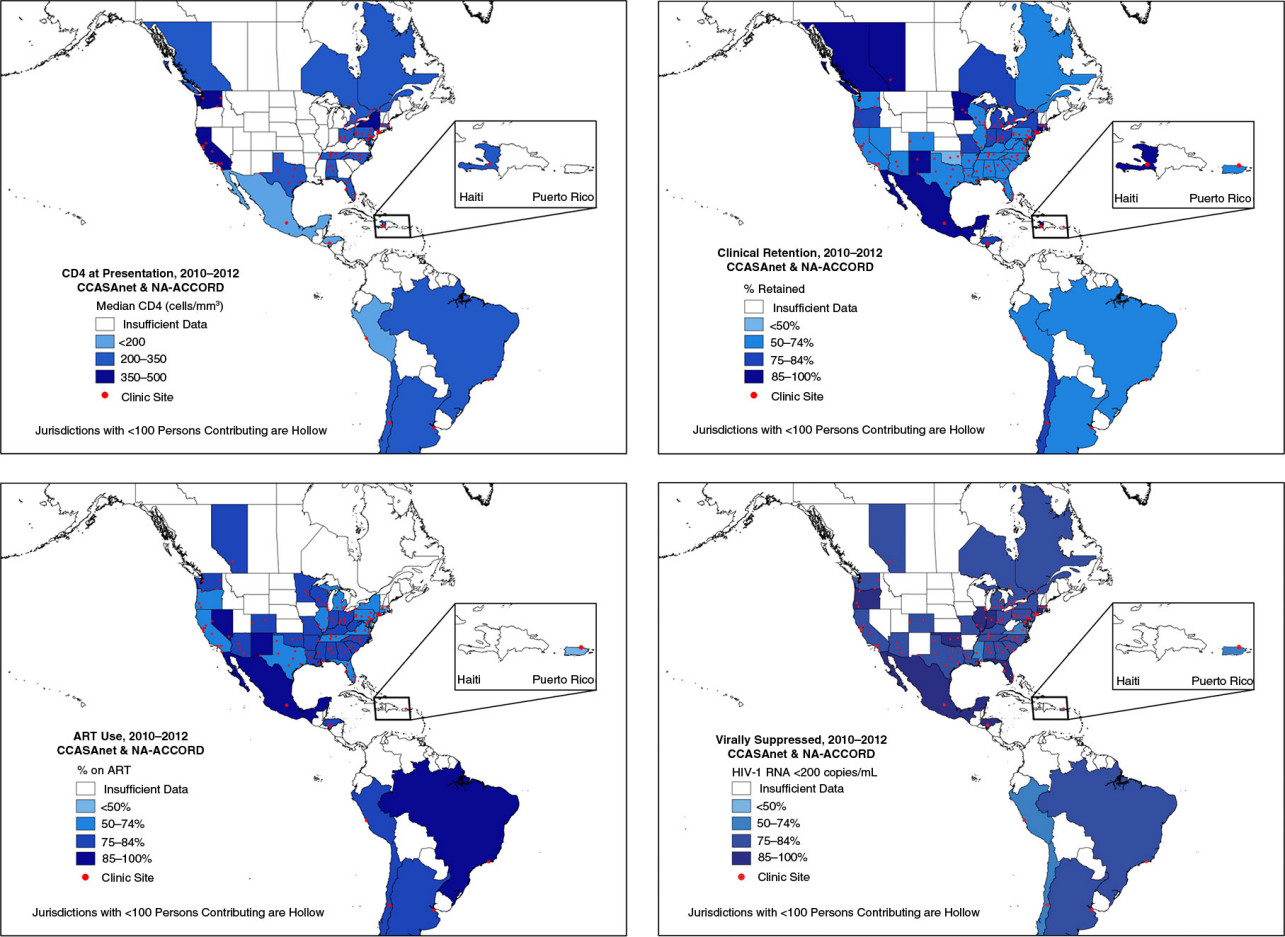
(a–d) Maps of four indicators of HIV care in North, Central, and South American Countries, NA-ACCORD and CCASAnet, 2010–2012. (a) Median CD4 count at presentation for HIV clinical care, defined as CD4 cell count within six months of entry into care at one of the participating clinical sites, among those who did not have a suppressed viral load or report previous ART use. (b) Percentage retained in HIV clinical care, defined as the percentage of patients with ≥1 encounter from 2010 to 2012 who had ≥2 HIV primary care visits >90 days apart (measured in the last year an individual contributes data from 2010 to 2012). (c) Percentage of HIV-positive patients prescribed antiretroviral therapy, defined as a the percentage with ≥1 HIV clinical encounter who were prescribed ≥3 antiretroviral agents from at least two classes or a triple nucleoside/nucleotide reverse transcriptase inhibitor regimen containing abacavir or tenofovir for ≥6 months in the last year they contributed from 2010 to 2012. (d) Percentage of HIV-positive patients with suppressed plasma HIV viral load, defined as the percentage of patients with ≥1 HIV clinical encounter and ≥1 HIV-1 plasma RNA measurement who had an HIV-1 RNA ≤200 copies/mL at their last measurement contributed in a calendar year from 2010 to 2012.

Geographical displays of medical data allow quick visualization of geographical associations and, because of this, can be more informative than other types of figures [1–3]. Maps enable visualization of gaps in the quality of care and serve as an important data source for monitoring geographically specific policies and programmes [4].

Many organizations currently produce maps of HIV indicators. Local maps of indicators for HIV testing and linkage of HIV-positive adults into care [5], state-level maps of the prevalence and incidence of HIV and other sexually transmitted infections [6], and country-level maps of demographic characteristics and other HIV indicators among HIV-positive adults are readily available [7–10]. These maps are accessible instruments for presenting important data to a broad audience of scientists, policy-makers, and the general public.

Monitoring trends in HIV clinical care indicators is essential to not only assessing the quality of HIV care, but also understanding the impact of suppressed HIV viral loads (VLs) on HIV transmission in a population [11,12]. In the context of changing health care policies and programmes (such as the Affordable Care Act in the United States [US] and the new global era of universal access to HIV treatment) capturing regional differences in indicators of HIV clinical care can inform our understanding of the impact of cost control and care improvement strategies.

To our knowledge, there are no publicly available, multi-country maps that focus on monitoring indicators of HIV clinical care among adults in care, perhaps due to the lack of consistent HIV population-based data across countries. Through the International Epidemiologic Databases to Evaluate AIDS (IeDEA) international research consortium, longitudinal clinical cohorts of adults in HIV care have harmonized their data across countries in seven geographical regions. Although these data are clinical care-driven and not population-based, they reflect adults receiving HIV care. Our objective was to create maps of 1) median CD4 cell count (CD4) at presentation for HIV care, 2) retention, 3) ART use and 4) HIV VL suppression indicators among adults in care for HIV infection, using data from the North American AIDS Cohort Collaboration on Research and Design (NA-ACCORD) and the Caribbean, Central and the South America network for HIV epidemiology (CCASAnet), both of which are regional collaborations of the IeDEA consortium.

## Methods

NA-ACCORD and CCASAnet are multisite collaborations of cohort studies of HIV-positive adults. Details on the NA-ACCORD and CCASAnet collaborations have been published previously [13,14]. Briefly, cohorts contribute data on patient demographic characteristics, prescribed antiretroviral therapy (ART), dates of primary HIV clinical visits, clinical diagnoses, laboratory test results (including CD4 count and HIV-1 RNA VL), and vital status. All data are transferred securely to the collaborations’ centralized data management cores, where they undergo quality control per a standardized protocol [15]. Participants were consented locally and the activities of the NA-ACCORD and CCASAnet have been reviewed and approved by the local institutional review boards for each site and at Johns Hopkins School of Medicine and Vanderbilt University School of Medicine, respectively.

We present a cross-sectional analysis using data contributed by clinical cohorts from 2010 to 2012. The 15 Canadian and US clinical cohorts included in this analysis had contributing clinical sites in four Canadian provinces and 48 US states (although participants resided in all 50 states), Washington DC, the Virgin Islands, and Puerto Rico, representing approximately 7.1% of persons known to be living with HIV in the US and Canada in 2012. The seven Caribbean, Central American, and South American clinical cohorts included had sites in the following countries: Argentina, Brazil, Chile, Haiti, Honduras, Mexico, and Peru, representing approximately 1.6% of persons known to be living with HIV in these countries. Geographical residence of participants was used to map the indicators; if this information was not available, the clinic location was used.

We estimated median CD4 at presentation for HIV clinical care, defined as a CD4 measured within six months of entry into care at one of the participating clinical sites, among those who successfully linked into care (defined as ≥2 HIV primary care visits within 12 months, >90 days apart) and did not have a suppressed VL or report previous ART use [16,17]. Additionally, we estimated the last three steps in the HIV Care Continuum (HIVCC): a) retention in care, defined among patients with ≥1 HIV primary care visits from 2010 to 2012 (excluding the year of death for patients who died) as the percentage of patients with ≥2 HIV primary care visits >90 days apart during the last calendar year that the patient contributed data [18]; b) ART use, defined as the percentage of patients with ≥1 visit who were prescribed ART for ≥6 months in the last year the patient contributed data from 2010 to 2012 [19]; c) VL suppression, defined as the percentage of patients with ≥1 visit who had a plasma HIV-1 RNA ≤200 copies/mL at their last measurement contributed in a calendar year from 2010 to 2012 [19]. Laboratory measures, namely CD4 count and HIV VL, were surrogate measures when HIV primary care visits were not available, which may result in a slight underestimation of retention in care [20]. ART was defined as a regimen of ≥3 antiretroviral agents from at least two classes or a triple nucleoside/nucleotide reverse transcriptase inhibitor regimen containing abacavir or tenofovir. To estimate the indicators with fidelity to their established definitions, a study population (defined by the denominator of the indicator) was selected for each indicator.

The indicators were summarized at the state- or territory-level in the United States, the province-level in Canada, and the country-level in the Caribbean, Central and South America. These summary data were then used to generate choropleth maps. Indicators for the location of the clinical sites were included on the maps. Data were summarized using SAS 9.3 (SAS Institute, Cary, NC), and maps were created using ArcGIS version 10.1 (Esri, Redlands, CA).

## Results

The study populations for each indicator were as follows: *N=*14,811 for median CD4 at presentation for care; *N*=87,979 for retention in care; *N*=84,757 for ART use; and *N*=51,118 for VL suppression (Table 1). The majority of participants (66–86%) in each of these populations were from the United States. The estimated indicators for Brazil, Chile, Haiti, Mexico, and Peru originated from one clinical cohort within those countries; many of these cohorts were multisite. Using the retention in care study population, the proportion of participants >50 years old was highest in the United States (51%), followed by Canada, Argentina, Brazil and Haiti (10–27%) and then Honduras, Mexico and Peru (8–9%). Median ages were 42–43 years in all countries except the United States (50 years), Canada (47 years), Mexico (39 years) and Peru (38 years). The country with the greatest proportion of women was Haiti (57%) followed by Honduras (47%). The United States had the greatest proportion of injection drug users (IDU) (20%). More than 40% of participants in Canada (47%), Chile (74%) and Mexico (67%) were men who have sex with men (MSM), but heterosexual contact was the primary transmission risk in Argentina (38%), Brazil (49%), Honduras (63%) and Peru (66%).

**Table 1:** Number of adults contributing to the specified HIV indicators, by country, NA-ACCORD and CCASAnet, 2010–2012

	CD4 at presentation for HIV care^a^		Retention in care^b^		ART use^c^		HIV-1 RNA suppression^d^	
Argentina	399	3%	2630	3%	2667	3%	2563	5%
Male	291	73%	1814	69%	1858	70%	1774	69%
≥ 50 yo	56	14%	720	27%	725	27%	706	28%
Brazil	400	3%	2735	3%	2809	3%	2768	5%
Male	274	69%	1734	63%	1791	64%	1759	64%
≥ 50 yo	42	11%	267	10%	736	26%	728	26%
Canada	976	7%	8104	9%	687	1%	1421	3%
Male	780	80%	6312	78%	557	81%	960	68%
≥ 50 yo	205	21%	1006	12%	327	48%	363	26%
Chile	527	4%	1705	2%	1969	2%	1797	4%
Male	476	90%	1509	89%	1744	89%	1591	89%
≥ 50 yo	62	12%	159	9%	466	24%	416	23%
Haiti	1493	10%	5271	6%	Not available		Not available	
Male	630	42%	2249	43%				
≥ 50 yo	238	16%	794	15%				
Honduras	129	1%	652	1%	449	1%	394	1%
Male	89	69%	344	53%	204	45%	171	43%
≥ 50 yo	20	16%	61	9%	67	15%	62	16%
Mexico	188	1%	739	1%	793	1%	785	2%
Male	166	88%	653	88%	702	89%	695	89%
≥ 50 yo	16	9%	57	8%	130	16%	127	16%
Peru	1014	7%	2070	2%	2576	3%	2555	5%
Male	766	76%	1420	69%	1821	71%	1796	70%
≥ 50 yo	119	12%	180	9%	401	16%	394	15%
USA	9685	65%	64,073	73%	72,807	86%	38,835	76%
Male	8239	85%	53,825	84%	61,312	84%	31,884	82%
≥ 50 yo	2886	30%	32,844	51%	36,865	51%	16,330	42%
Total	14,811	100%	87,979	100%	84,757	100%	51,118	100%

yo=years old.

The percentages listed for a country are the proportion of the total study population contributed by that country's cohorts. The percentages listed for “Male” and “≥50 yo” are the proportion of the study population *within countries* that are male and ≥50 yo.

a Presentation was defined as enrolment in a participating cohort between 2010 and 2012 and ≥1 CD4 within six months of enrolment.

b Retention in care was measured using data from clinical HIV primary care visits, with the exception of Argentina, Peru, and the Canadian province of British Columbia (Canada), for which retention was measured using CD4 or VL measures as proxies for visits; the denominator was defined as those who had ≥1 visit in the study period.

c The denominator was defined as those who had ≥1 visit in the study period.

d The denominator was defined as those who had ≥1 visit and ≥1 HIV-1 RNA measurement in the study period.

Mexico (127 cells/mm^3^), Honduras (163 cells/mm^3^) and Peru (175 cells/mm^3^) were the only countries with a median CD4 at HIV care presentation <200 cells/mm^3^; all other states, provinces and countries had a median CD4 ≥200 cells/mm^3^ at presentation for care (Figure 1a). Only three US states and the District of Columbia had a median CD4 at presentation >350 cells/mm^3^. Retention in care varied from 55 to 94%, with lower (50–74%) retention in the US West, South and Mid-Atlantic, as well as in Argentina, Brazil and Peru (Figure 1b). Haiti, Mexico and several states had >85% retention in care. ART use was highest (90%) in Mexico (Figure 1c). The percentages of patients with suppressed plasma HIV-1 RNA in the southern and north-eastern United States were lower than in most of Central and South America (Figure 1d). Estimates for each indicator at the state, province and country level can be found in the Supplementary file.

Animated and static maps with options for stratification that display these indicators from 2000 to 2012 are available at www.naaccord.org.

## Discussion

Mapping HIV indicators in North, Central and South America provides value to HIV researchers, epidemiologists, and programme and policymakers by 1) supporting research through the visualization of gaps in the quality of HIV care at the national level and by allowing for comparison between countries [4]; 2) allowing for monitoring of country-level policy and programme goals; and 3) providing estimates for quantitative models of HIV care. Maps of HIV indicators measured longitudinally among adults who have successfully linked to HIV care complement current efforts to geographically visualize other HIV indicators from population-based surveillance data and surveys [5–10].

Although the definitions of the HIV clinical care indicators we have presented were formalized in the United States, we show their utility to monitor progress in the HIVCC across the Western Hemisphere. Using these specific indicators to monitor HIV care in any given region is contingent upon whether these indicators reflect clinical care expectations in the region. Three of the four indicators mapped in this report have been endorsed for use to monitor progress towards US National HIV Strategy goals [21]. The retention in care indicator employed was endorsed by the US Institute of Medicine [18]. We have previously shown that this measure may slightly overestimate (by approximately 5%) the proportion of patients retained in care defined using the US Department of Health and Human Services (DHHS) definition and is better suited to measure trends over time [22]. The indicators for the proportion of patients prescribed ART and the proportion with suppressed HIV VL are endorsed by DHHS [19], but laboratory measures were surrogate measures of clinical visits when these data were not available. The median CD4 cell count at presentation indicator is a useful measure of immunologic status at entry into care and has been previously monitored in the NA-ACCORD [16,17].

Audiences for these maps may include government agencies, non-government and non-profit organizations, academic researchers, public health workers and officials, and industry. Easy-to-access geographical displays of HIV indicators can aid organizations with programme decision-making and policy. The consistent measurement of the specified indicators allows for direct comparison across states, provinces, regions and countries (assuming the estimates are generalizable within each region). Indicators can be employed over time to monitor progress towards HIV policy or programme goals endorsed by countries, or by international organizations, assuming the data required to measure the indicators are available cross-nationally. Additionally, estimates of HIV indicators are needed by researchers to understand the burden and the changing aspects of the HIV epidemics within geographical borders. Such estimates are particularly valuable for inputs informing models of cost-effectiveness of HIV interventions [23].

Alternatively, in communicating the changing nature of health care access and quality to the general public through the lens of policy and programme changes (e.g. altered trajectory of US National HIV Strategy goal indicators after expansion of publically funded health insurance programmes within the US), policy makers and scientists may employ many data visualization techniques, among which maps may be particularly engaging. Whether demonstrating need through mapping underperforming regions, such as increased HIV-related mortality throughout the American South [24], or demonstrating success through mapping the impact of successful regional interventions, maps are easily understood and convey a large amount of information. Dissemination of high quality analyses and evidence through the maps may impact the lives of persons living with HIV infection and alter the epidemiology of the epidemic by providing convincing visual evidence to motivate improvement of HIV health care services and the political will to implement needed changes.

The maps created here are possible because of the collaboration of the NA-ACCORD and CCASAnet. A standard data protocol is employed to merge data, even though the data originate from diverse clinical settings with varying levels of resources [15]. A limitation of these data, however, is the extrapolation of HIV clinical care indicator estimates from patients in the contributing clinical cohorts to HIV-positive adults in HIV care within a region, particularly in regions in which the data are from one single-site clinical cohort. Because these data are from HIV clinics and not population-based, comparisons of people in HIV clinical care in a given region with participants in our contributing HIV clinical cohorts are needed to determine generalizability of the indicators to all HIV-positive persons in HIV care in a given region. Although the NA-ACCORD population has been shown to have similar demographic characteristics as compared to persons living with HIV in the United States, comparable data do not exist for other IeDEA regions [25]. An additional important limitation is that the denominators of the retention in care, ART use and viral suppression indicators do not necessarily represent all HIV-positive persons in care, but rather include those in “active care” defined as those having ≥1 HIV primary care visit. For example, if a person was seen in the clinic in 2009, but not in 2010, that individual is not included in the retention in care indicator. The CD4 at presentation for care indicator is among those who successfully link into care. Potential underestimation of the proportion retained in care could result from our inability to determine if a participant transferred care, as opposed to falling out of care. Finally, a few of the individual participating HIV clinical cohorts have previously estimated some of these indicators, but there were differences in the selection criteria and indicator definitions used compared to this presented work.

In order for maps to be useful, the data collected must be representative of the region and analyzed in a timely fashion to have the biggest impact. Given the enormous effort needed to collect, clean and standardize the data, a delay of 2–4 years is expected. To improve the speed at which these data become available, annual updates of the maps (with interactive features that allow for stratification by demographic characteristics) will be posted at www.naaccord.org.

By mapping harmonized data and standardized definitions of HIV indicators across North, Central and South America, geographical associations of these HIV indicators are readily apparent. Although these mapped estimates are not population-based, they are useful tools for implementation science, monitoring progress towards geographically specified policy and programme goals, and serving as inputs for cost-effectiveness models.

## Supplementary Material

A picture is worth a thousand words: maps of HIV indicators to inform research, programs, and policy from NA-ACCORD and CCASAnet clinical cohorts
