# Supplementary material for: A picture is worth a thousand words: maps of HIV indicators to inform research, programs, and policy from NA-ACCORD and CCASAnet clinical cohorts
Source: J Int AIDS Soc. 2016 Apr 4;19(1):20707. doi: 10.7448/IAS.19.1.20707 (PMC4821890; doi:10.7448/IAS.19.1.20707)
Supplement: A picture is worth a thousand words: maps of HIV indicators to inform research, programs, and policy from NA-ACCORD and CCASAnet clinical cohorts [file JIAS-19-20707-s001.pdf]

ADDITIONAL FILES

Supplemental Table 1. Complete distributions of CCASAnet and NA-ACCORD clinical populations contributing to HIV indicators by country, state, territory, and province mapped.

Jurisdiction	Median CD4 at Entry	N=14,811	(%)	Percent Retained	N=87,979	(%)	Percent on ART	N=84,757	(%)	Percent Virally Suppressed	N=51,118	(%)
**Argentina**	284	399	(3)	60	2,630	(3)	83	2,667	(3)	79	2,563	(5)
**Brazil**	253	400	(3)	75	2,735	(3)	86	2,809	(3)	79	2,768	(5)
**Chile**	267	527	(4)	76	1,705	(2)	78	1,969	(2)	74	1,797	(4)
**Haiti**[a]	216	1493	(10)	86	5,271	(6)	*	0	(0)	*	0	(0)
**Honduras**	163	129	(1)	78	652	(1)	84	449	(1)	87	394	(1)
**Mexico**	127	188	(1)	87	739	(1)	90	793	(1)	88	785	(2)
**Peru**	175	1014	(7)	66	2,070	(2)	77	2,576	(3)	71	2,555	(5)
**United States**	341	9685	(65)	73	64,073	(73)	67	72,808	(86)	78	38,835	(76)
**Northeast**												
**CT**	*	†	(0)	89	187	(0)	74	211	(0)	93	121	(0)
**MA**	342	143	(1)	79	1,529	(2)	29	1,727	(2)	79	717	(1)
**ME**	*	†	(0)	*	61	(0)	*	63	(0)	*	17	(0)
**NH**	*	†	(0)	*	41	(0)	*	47	(0)	*	18	(0)
**NJ**	*	14	(0)	77	484	(1)	68	529	(1)	83	279	(1)
**NY**	400	1068	(7)	84	7,063	(8)	52	7,538	(9)	77	4,220	(8)
**PA**	332	302	(2)	73	3,060	(4)	59	3,338	(4)	77	1,914	(4)
**RI**	*	†	(0)	*	64	(0)	*	74	(0)	*	39	(0)
**VT**	*	0	(0)	*	15	(0)	*	19	(0)	*	†	(0)
**Midwest**												
**IA**	*	†	(0)	*	77	(0)	*	81	(0)	*	33	(0)
**IL**	*	53	(0)	73	1,507	(2)	71	1,593	(2)	87	931	(2)
**IN**	*	†	(0)	78	257	(0)	81	272	(0)	88	134	(0)
**KS**	*	†	(0)	*	69	(0)	*	71	(0)	*	37	(0)
**MI**	*	†	(0)	75	378	(0)	73	410	(1)	78	196	(0)
**MN**	*	†	(0)	94	125	(0)	82	140	(0)	*	53	(0)
**MO**	*	†	(0)	76	294	(0)	79	322	(0)	80	151	(0)
**ND**	*	0	(0)	*	16	(0)	*	16	(0)	*	†	(0)
**NE**	*	†	(0)	*	59	(0)	*	67	(0)	*	64	(0)
**OH**	313	115	(1)	77	1,497	(2)	76	1,638	(2)	77	899	(2)

**SD**	*	†	(0)	*	29	(0)	*	30	(0)	*	13	(0)
**WI**	*	†	(0)	70	110	(0)	78	121	(0)	*	93	(0)
**South**												
**AL**	319	483	(3)	72	2,300	(3)	77	2,533	(3)	79	1,639	(3)
**AR**	*	†	(0)	68	185	(0)	77	197	(0)	*	92	(0)
**DC**	368	126	(1)	69	1,192	(1)	70	1,293	(2)	73	528	(1)
**DE**	*	0	(0)	*	78	(0)	*	85	(0)	*	37	(0)
**FL**	288	394	(3)	73	4,531	(5)	63	4,857	(6)	86	3,075	(6)
**GA**	*	56	(0)	68	1,374	(2)	76	1,474	(2)	79	812	(2)
**KY**	*	10	(0)	72	267	(0)	80	282	(0)	82	153	(0)
**LA**	*	11	(0)	63	365	(0)	76	406	(1)	76	209	(0)
**MD**	283	481	(3)	71	3,563	(4)	72	4,053	(5)	79	2,226	(4)
**MS**	*	10	(0)	64	267	(0)	79	284	(0)	69	124	(0)
**NC**	300	196	(1)	73	2,186	(3)	83	2,389	(3)	80	1,259	(3)
**OK**	*	†	(0)	21	179	(0)	83	194	(0)	95	102	(0)
**SC**	*	10	(0)	65	488	(1)	76	536	(1)	79	274	(1)
**TN**	314	337	(2)	75	2,906	(3)	70	3,282	(4)	78	1,960	(4)
**TX**	233	871	(6)	74	7,070	(8)	75	7,469	(9)	76	4,620	(9)
**VA**	327	118	(1)	67	932	(1)	75	1,029	(1)	75	366	(1)
**WV**	*	†	(0)	55	115	(0)	80	124	(0)	*	40	(0)
**West**												
**AK**	*	0	(0)	*	†	(0)		†	(0)	*	†	(0)
**AZ**	*	†	(0)	73	456	(1)	8	495	(1)	85	196	(0)
**CA**	362	1033	(7)	69	10,119	(12)	68	11,029	(13)	78	4,504	(9)
**CO**	*	55	(0)	71	1,046	(1)	75	1,074	(1)	80	406	(1)
**HI**	*	0	(0)	*	†	(0)	*	†	(0)	*	†	(0)
**ID**	*	0	(0)	*	43	(0)	*	45	(0)	*	16	(0)
**MT**	*	0	(0)	*	†	(0)	*	†	(0)	*	†	(0)
**NM**	*	0	(0)	88	106	(0)	87	111	(0)	*	45	(0)
**NV**	*	10	(0)	55	260	(0)	89	285	(0)	*	85	(0)
**OR**	*	99	(1)	77	798	(1)	59	840	(1)	87	392	(1)
**UT**	*	†	(0)	*	64	(0)	*	68	(0)	*	43	(0)
**WA**	352	140	(1)	68	1,805	(2)	79	1,892	(2)	76	877	(2)
**WY**	*	0	(0)	*	10	(0)	*	12	(0)	*	†	(0)
**Puerto Rico**	*	12	(0)	73	576	(1)	48	692	(1)	71	331	(1)
**Virgin Islands**	*	0	(0)	*	†	(0)	*	†	(0)	*	†	(0)

**Canada**	285	976	(7)	85	8,104	(9)	79	687	(1)	77	1,413	(3)
**East**												
**NB**	*	0	(0)	*	0	(0)	*	0	(0)	*	0	(0)
**NL**	*	0	(0)	*	0	(0)	*	0	(0)	*	0	(0)
**NS**	*	0	(0)	*	0	(0)	*	0	(0)	*	0	(0)
**ON**[a]	300	214	(1)	77	1555	(2)	*	0	(0)	77	659	(1)
**PE**	*	0	(0)	*	†	(0)	*	0	(0)	*	0	(0)
**QC**	291	106	(1)	71	1,441	(2)	*	0	(0)	77	551	(1)
**West**												
**AB**	*	87	(1)	86	627	(1)	79	677	(1)	75	202	(0)
**BC**[a]	280	555	(4)	91	4441	(5)	*	†	(0)	*	†	(0)
**MB**	*	0	(0)	*	0	(0)	*	0	(0)	*	0	(0)
**SK**	*	0	(0)	*	†	(0)	*	†	(0)	*	0	(0)
**Northern**												
**NT**	*	0	(0)	*	†	(0)	*	0	(0)	*	0	(0)
**NU**	*	0	(0)	*	0	(0)	*	0	(0)	*	0	(0)
**YK**	*	0	(0)	*	†	(0)	*	0	(0)	*	0	(0)

a. Cohorts representing these countries and provinces either did not contribute data for indicators with missing values, or else used ART receipt as an inclusion criterion and therefore do not contribute to the ART use or viral suppression indicators.

* <100 individuals contributing in the respective country, state, or province, reflecting an inadequate population to produce a stable estimate of the indicator.

† 1-10 individuals contributing in the respective country, state, or province; the exact count has been omitted to avoid inadvertent disclosure.
